# Supplementary material for: Heparan Sulfate: A Regulator of White Adipocyte Differentiation and of Vascular/Adipocyte Interactions
Source: Biomedicines. 2022 Aug 29;10(9):2115. doi: 10.3390/biomedicines10092115 (PMC9495464; doi:10.3390/biomedicines10092115)
Supplement: Supplementary file 1 [file biomedicines-10-02115-s001.zip › biomedicines-1824327-supplementary.pdf]

Supplementary Materials:

Table S1. Examples of Adipokines that possess HB-domain.

**Fibroblast growth factor-1**	**Fibroblast growth factor-2**	**Vascular endothelial growth factor A_{165}**	**Vascular endothelial growth factor A_{189}**
Placental growth factor	Heparan binding epithelial growth factor	Hepatocyte growth factor	Transforming growth factor-β
Interferon γ	Platelet derived growth factor-BB	Platelet derived growth factor AA	Pleotrophin
Platelet factor 4	Interleukin 8	Macrophage inflammatory protein 1	Interleukin 10
